# Molecular-genetic causes for the high frequency of phenylketonuria in the population from the North Caucasus

**DOI:** 10.1371/journal.pone.0201489

**Published:** 2018-08-01

**Authors:** Polina Gundorova, Rena A. Zinchenko, Irina A. Kuznetsova, Elena A. Bliznetz, Anna A. Stepanova, Aleksander V. Polyakov

**Affiliations:** 1 Laboratory of DNA-diagnostics, Federal State Budgetary Institution "Research Centre for Medical Genetics", Moscow, Russia; 2 Laboratory of Genetic Epidemiology, Federal State Budgetary Institution "Research Centre for Medical Genetics", Moscow, Russia; 3 Pirogov Russian National Research Medical University, Moscow, Russia; University of Innsbruck, AUSTRIA

## Abstract

Phenylketonuria is an inherited disease caused by mutations in the phenylalanine hydroxylase gene *PAH*. Different *PAH* pathogenic variants occur in different ethnic groups with various frequencies and the incidence of the disease itself varies from country to country. In the Caucasus region of Russia, some ethnoses are geographically and culturally isolated from each other. The tradition of monoethnic marriages may cause decreased genetic variability in those populations. In the Karachay-Cherkess Republic (Russia), the highest incidence of phenylketonuria in the world has been detected (1:850 newborns) in the region and 1:332 among the titular nation Karachays. Here, we showed that this phenomenon is due to the widespread prevalence of the p.Arg261* variant. Its allele frequency among Karachay patients with PKU was 68.4% and the carrier frequency in Karachays was 1:16 healthy individuals. *PAH* haplotype analysis showed a unique common origin. The founder haplotype and mutation “age” were estimated by analyzing the linkage disequilibrium between p.Arg261* and extragenic short tandem repeat loci. The p.Arg261* variant occurred in the Karachays population 10.2 ± 2.7 generations ago (275 ± 73 years) and its spread occurred in parallel with the growth of the population.

## Introduction

An increase in the concentration of phenylalanine (Phe) above the physiological norm is called hyperphenylalaninemia (HPA). The main type of HPA is phenylketonuria (PKU; OMIM #261600). Phenylketonuria is an autosomal recessive inherited error of metabolism resulting from a deficiency in phenylalanine hydroxylase (EC 1.14.16.1), an enzyme that catalyzes the hydroxylation of phenylalanine to tyrosine, the rate-limiting step in phenylalanine catabolism [1]. Phenylketonuria is the most widespread aminoacidopathy in the world. The disease incidence is about 1:10000 worldwide and about 1:7000 in Russia [2]. PKU is included in neonatal screening programs, which have been conducted in Russia since 1991. The highest PKU incidences were detected in Turkey at 1:4000 [3] and Northern Ireland at 1:6500 [4]. According to the clinical classification, classical PKU is characterized by a Phe concentration above 20 mg/dl, moderate PKU from 10–20 mg/dl, and mild HPA (mHPA) from 2–10 mg/dl [5]. In this work, we use the term “mHPA” to emphasize the mild clinical diagnosis, while the term “PKU” is used to emphasize severe clinical features. “HPA” is used to describe the disease in general.

Since Russia is a multinational country, frequencies of monogenic diseases and different pathogenic variants can differ drastically among members of different ethnic groups [6]. The Karachay-Cherkess Republic (KCR) is a region in the Southwest of the Russian Federation located in the north of the Caucasus Mountains. Karachay-Cherkessia has a population of 469,000 people, 43% of which live in cities. There are four major nationalities in the region: Karachays (41%), Russians (32%), Cherkessians (12%), and Abazins (8%). In two cities of the region, Karachaevsk and Cherkessk, representatives of these groups live together, but in the rural districts, ethnoses are largely isolated from each other. Monoethnic marriages between Karachays, according to epidemiological studies, account for 88.3% of marriages [7]. In a small population, this situation can decrease genetic variability and change the frequency of homozygotes in the population; in particular, an increase in the frequencies of specific nosological forms is possible.

In Karachay-Cherkessia, patients with PKU and mHPA were identified and examined. Interestingly, the p.Arg261* (c.781C>T) *PAH* variant is very widespread among them. The allele frequency of p.Arg261* is 68.4% among PKU patients and 32.5% among mHPA patients [8, 9], so it is the predominant variant in this region. The presence of those genetic peculiarities in Karachay-Cherkessia presumably results from reproductive isolation and genetic drift in populations residing on this territory.

## Materials and methods

### Patients

DNA of 26 Karachays with a PKU diagnosis and a homozygous p.Arg261* genotype and DNA of 33 healthy relatives was used for haplotype analysis. The written informed consent for biological material collection, research, and publication of their results in the press was obtained. In case of children aged under 18, written informed consent was obtained from parents. Patients with PKU were selected through the regional medical-genetic counseling center. Ethics Committee of Federal State Budgetary Institution "Research Centre for Medical Genetics" had approved the study with decision of Protocol No. 2 at the meeting of March 15, 2017.

Expeditions for the purpose of collecting the material were carried out by the staff of the Laboratory of Genetic Epidemiology of the FSBI "Research Centre for Medical Genetics" in the period from 2013 to 2016. Patient selection was carried out through the regional medical-genetic counseling center of the Karachay-Cherkess Republic. As a result of the expedition work, families with a medical history of diseases "PKU" and "mHPA" were identified. For the haplotype analysis, only families with homozygous p.Arg261* genotype probands were selected.

### Population sample

Due to expedition work in the Karachaevsk and Cherkessk cities and six districts of the KCR, biological material was obtained from 676 healthy residents. The involvement of participants in the study was carried out through the regional medical-genetic counseling center of the KCR. Healthy residents were invited for the diagnosis of various inherited diseases such as HPA, cystic fibrosis, and hearing loss. Volunteers were further selected according to the following criteria: healthy for somatic and hereditary diseases; not related individuals; Karachay, Circassian, Abazin, or Nogay heritage up to the third generation; and natives of a particular region. Written informed consent was obtained for biological material collection, research, and publication of their results in the press. All volunteers were examined visually for the exclusion of hereditary diseases and syndromes by clinical genetic physicians and life-long anamnesis was collected. Study participants’ ages were between 18–40 years.

### *PAH* variant detection

All *PAH* gene variants in the paper are related to the reference sequence NM_000277.1. A custom allele-specific MLPA panel with polyacrylamide electrophoresis visualization was created earlier based on the PKU mutation spectrum in the KCR [9]. *PAH* variants p.Arg413Pro (c.1238G>C), p.Arg408Trp (c.1222C>T), p.Phe331Ser (c.992T>C), p.Arg261* (c.781C>T), p.Pro211Thr (c.631C>A), and p.Pro211Leu (c.632C>T) were studied in healthy Karachay-Cherkessia residents.

### p.Arg261* chromosome haplotype investigation

Haplotype analysis was performed by studying seven intragenic single nucleotide polymorphism (SNP) loci (restriction fragment length polymorphism [RFLP] haplotypes), as well as intragenic tandem repeats regions via variable number tandem repeats (VNTRs) and short tandem repeats (STRs). SNPs were detected using the multiplex ligation-dependent probe amplification (MLPA) method visualized by acrylamide electrophoresis (S1 Appendix). The copy number analysis of the *PAH* gene intragenic tandem STRs and VNTRs was carried out by polymerase chain reaction (PCR) and Sanger sequencing [10, 11]. EcoRV restriction site analysis was not conducted due to technical limitations.

The analysis of the extragenic STR loci D12S1588, D12S1727, D12S78, D12S338, and D12S317 was carried out by PCR and polyacrylamide electrophoresis. The analysis was performed on the DNA of patients homozygous for the variant p.Arg261*, the DNA of all their relatives whose material was available for research, and also in the population sample of the Karachays.

### Linkage disequilibrium

To identify alleles associated with PKU, we analyzed polymorphic markers and determined the haplotypes of chromosomes bearing the p.Arg261* variant. When comparing the allele frequencies on chromosomes with a pathogenic variant and on population sample chromosomes, the *χ*^*2*^ criteria was used. For estimating the linkage disequilibrium of polymorphic markers in the patient group, we calculated δ [12]

$$\delta =\frac{P_{D}-P_{N}}{1-P_{N}}$$ (1)

where *δ* is a linkage disequilibrium, P_D_ is the frequency of the associated allele among chromosomes with a pathogenic variant, and P_N_ is the frequency of the same allele among normal chromosomes. The confidence interval for *δ* was estimated as follows [13]: as *δ* is a function of the ratio of two independent random variables, the variance can be approximated as

$$var(\delta )=\frac{1}{{\left(1-P_{N}\right)}^{4}}\sigma_{pN}^{2}(\sigma_{pD}^{2}+{\left(1-P_{D}\right)}^{2})+\frac{\sigma_{pD}^{2}}{{\left(1-P_{N}\right)}^{2}}.$$ (2)

In formula (2), σ^2^_*p*N_ = P_N_ (1 –P_N_)/n_N_ and σ^2^_*p*D_ = P_D_ (1 –P_D_)/n_D_, *n*_*N*_ and *n*_*D*_−the sizes of samples of mutant and normal chromosomes. The corresponding 95% confidence interval for *δ* can be obtained as δ ± 2σ_δ_, while σ_δ_^2^ = var(δ) [13, 14].

### Mutation “age”

The determination of mutation “age” is possible in cases where a mutation has spread as a result of the founder effect. The founder's chromosome bearing a new variant appears in the population by migration or *de novo* via mutation. Then, from generation to generation, the proportion of mutant chromosomes with the founder's haplotype decreases.

$$g=\frac{lg\frac{1-Q}{1-P_{N}}}{lg(1-\theta )}$$ (3)

where *g* is the number of generations; *Q* is the proportion of mutant chromosomes without the founder's haplotype allele; *P*_*N*_ is the frequency of the founder's allele in the population; and *θ* is the recombination fraction. At genetic distances less than 10 cM, *θ* is equal to the genetic distance in Morganids [15].

## Results

### Definition of the estimated incidence of HPA and carrier frequency among healthy residents

Newborn screening data show the incidence of HPA in the KCR as 1:850 newborns [8]. To calculate the PKU incidence in different ethnic groups, we performed DNA analysis among healthy residents. The custom *PAH* mutation-detecting panel was designed for DNA diagnostics for KCR residents. Six *PAH* variants can be detected by this method: p.Arg413Pro, p.Arg408Trp, p.Phe331Ser, p.Arg261*, p.Pro211Thr, and p.Pro211Leu. The overall allele frequency (calculated among patients) is 76.8% for KCR residents in general and 81.4% for Karachays [9]. Carrier frequency and disease incidence were calculated, taking into account the effectiveness of the method.

Healthy indigenous representatives of ethical groups were examined: 328 Karachays, 104 Circassians, 126 Abazins, and 118 Nogays. Thirty-eight carriers of *PAH* variants were identified among them (Table 1). The majority of carriers identified were among Karachays, and this is consistent with the data of PKU patients. The calculated carrier frequency was 1:9 healthy Karachays and PKU incidence was 1:332 Karachays. For a small population, this is a catastrophically high carrier frequency and monogenic disease incidence. The calculated PKU incidence for Circassians and Nogays was 1:6380 and 1:8213, respectively, which was similar to the mean value in Russia. The calculated PKU incidence for Abazins was 1:4162, which was above the average.

**Table 1:** Survey data of healthy KCR residents.

Characteristic / Nationality	Karachays	Circassians	Abazins	Nogays
Number of examinees	328	104	126	118
Carriers identified	31	2	3	2
Carrier frequency ^a^	1:9	1:40	1:32	1:45
PKU incidence ^b^	1:332	1:6380	1:4162	1:8213
PKU incidence per 1000, ‰	3,01±0.10	0.16±0.04	0.24±0.04	0.12±0.03

^a^ The proportion of *PAH* variant carriers identified in the studied groups of indigenous representatives of each ethnic group, taking into account the total allele frequency of variants studied.

^b^ PKU incidence among newborns

The p.Arg408Trp variant, which is predominant in Caucasians including Russians, was not found in Karachays at all, but it was found in Circassians, Nogays, and once in Abazins. The carrier frequency of p.Arg261* among Karachays was 1:16. It is obvious that the high incidence of PKU in the Karachay-Cherkess Republic is provided mainly by the high heterozygous carrier frequency of *PAH* gene variants among the Karachays, mainly by the p.Arg261* mutation.

### Features of the major variant p.Arg261* spread among Karachays

The wide distribution of the variant p.Arg261* among Karachays suggests the presence of the founder effect. When a new allele occurs, there is complete linkage disequilibrium of this locus with the rest. As a result of recombination, this imbalance decreases from generation to generation. This effect could be used for determining the moment when variant occurred or the mutation “age” [16–18].

DNA samples of 26 patients with PKU that were p.Arg261* homozygous were analyzed using RFLP for *PAH* haplotypes [19], a historically established method for analyzing the origin of *PAH* variants. The same homozygous haplotype was identified in all p.Arg261* homozygous patients. The haplotype identified for the p.Arg261* variant was similar to 8, 10, or 41 *PAH* haplotypes: PvuIIa +, PvuIIb -, BglII -, XmnI -, MspI +, EcoRI +, AluI +, STR 240 bp, VNTR 7 monomers. Since identical alleles for all the markers investigated were identified on all studied chromosomes with the p.Arg261* variant, linkage disequilibrium was δ = 1, and the haplotype was linked to the variant.

An analysis of the linkage with polymorphic markers lying at different genetic distances from the gene can reveal the degree of the haplotype decay and the mutation “age”. A study of genotypes of chromosomes was carried out on five STR markers located near the *PAH* gene in two samples: 26 Karachays homozygous for p.Arg261* and 30 healthy Karachays who were not related to the PKU patients or p.Arg261* carriers. Five STR markers located in the interval of 5002 kb around the *PAH* gene with high heterozygosity were selected. The frequencies of the identified alleles are presented in S2 Appendix.

For each allele of all the markers studied, the value of the linkage disequilibrium δ was determined using the formula (1). Using the obtained data, the allelic composition of the “founder haplotype” was revealed. Since at the time when the variant occurs, a complete disequilibrium with the marker alleles is formed, the “founder haplotype”, and as a result of recombination this disequilibrium decays with time, at the present moment the alleles that have the maximum disequilibrium coincide with the “founder haplotype”. Therefore, in determining the ancestral alleles, the greatest positive values of δ were taken into account (Table 2). The most likely “founder haplotype” was D12S1588-D12S1727-D12S78-D12S338-D12S317: 5-8-8-1-16.

**Table 2:** Linkage disequilibrium analysis between the p.Arg261* variant in Karachays and microsatellites close to the *PAH* gene.

Marker	Allele	θ, сМ	χ2	δ± 95%Cl	g	g (mean)
D12S1588	5	4.82	30.2	0.58±0.16	11.08	10.2±2.7
D12S1727	8	2.81	34.1	0.71±0.15	12.24	
*РАН*	p.Arg261*					
D12S78	8	1.87	93.0	0.92±0.08		
D12S338	1	1.87	64.5	0.93±0.08		
D12S317	16	4.28	66.4	0.73±0.12	7.17	

Data shown in Table 2 show that the value of the linkage disequilibrium decreased with increasing distance from the *PAH* gene. This pattern is typical for populations in which the distribution of the allele occurred as a result of the founder effect. High values ​​of linkage disequilibrium were maintained at a rather large distance from the gene: 4.82 cM toward the centromere and 4.28 cM toward the telomeres. This may indicate a relatively low age of the founder effect in the studied population.

Haplotypes of chromosomes with the p.Arg261* variant are presented in Table 3. In heterozygous patients, haplotypes were determined using family analysis where DNA from parents and other relatives was available (families No. 13, 21, 29, 32, 36, 37, 39, 40, 47, 49, 66, 109, 110, 111, and 201). In cases where it was not possible to determine on which chromosome one or another allele is located, the variants were determined through a fractional line (families No. 53 and 64).

**Table 3:** Haplotypes of p.Arg261*-bearing chromosomes at five microsatellite loci.

Marker	D12S1588	D12S1727	*PAH*	D12S78	D12S338	D12S317
сМ^a^	105.18	107.19	110	111.87	111.87	114.28
Mb^b^	100.594	101.701	103.2715	104.264	104.541	105.596
Family №						
21	5^c^	8	m	8	1	16
32	5	8	m	8	1	16
35	5	8	m	8	1	16
35	5	8	m	8	1	16
36	5	8	m	8	1	16
37	5	8	m	8	1	16
39	5	8	m	8	1	16
40	5	8	m	8	1	16
40	5	8	m	8	1	16
41	5	8	m	8	1	16
42	5	8	m	8	1	16
42	5	8	m	8	1	16
43	5	8	m	8	1	16
43	5	8	m	8	1	16
45	5	8	m	8	1	16
45	5	8	m	8	1	16
46	5	8	m	8	1	16
46	5	8	m	8	1	16
48	5	8	m	8	1	16
49	5	8	m	8	1	16
54	5	8	m	8	1	16
54	5	8	m	8	1	16
109	5	8	m	8	1	16
13	5	8	m	8	1	16
20.1	5	8	m	8	1	16
48	5	8	m	8	1	13
47	5	8	m	8	1	4
111	5	8	m	8	1	4
111	5	8	m	8	1	4
29	5	8	m	8	1	3
66	5	8	m	8	1	3
53	2/4	8	m	8	1	16
49	4	8	m	8	1	16
13	4	8	m	8	1	16
37	3	8	m	8	1	16
110	3	8	m	8	1	16
20.1	4	8	m	8	1	16
66	3	8	m	8	1	3
32	5	11	m	8	1	16
21	3	7	m	8	1	16
36	3	7	m	8	1	16
53	2/4	7	m	8	1	16
109	3	7	m	8	1	16
30	3	11	m	8	1	15
39	3	11	m	8	1	14
30	2	11	m	8	2	15
64	2/4	11	m	8	2	14/16
64	2/4	8	m	8	2	14/16
29	5	8	m	12	1	3
47	3	10	m	3	1	4
110	2	11	m	1	1	14
41	2	11	m	1	1	16

^a^ The distance between the STR-marker and the *PAH* gene in centimorgans.

^b^ The distance between the STR-marker and the *PAH* gene in megabases.

^c^ The saved fragments of the Karachay “founder haplotype” are highlighted by filling.

The presence of a repeating haplotype in chromosomes with p.Arg261* confirmed the assumption that the variant was widely spread among the Karachays as a result of the founder effect. Calculation of the p.Arg261* mutation “age” was carried out according to formula (2) for markers D12S1588, D12S1727, and D12S317. For markers D12S78 and D12S338, the value of linkage disequilibrium with the confidence interval includes the value of δ = 1, that is, a small number of recombination events are detected for these markers, and they are actually still linked to the variant. For this reason, calculating the mutation “age” by markers D12S78 and D12S338 would be invalid.

The calculated mutation “age” for markers D12S1588, D12S1727, and D12S317 is presented in Table 2. The average value of the generations passed since the beginning of the variant spread was g = 10.2 ± 2.7 generations. The average age of one generation is calculated as the average value of the period from the birth of the parent to the birth of the first and last child. At the same time, the average age of a generation in females is always less than that in males. Studies conducted by anthropologists in primitive lifestyle populations determined the average age of one generation to be 30 years [14]. According to a study conducted in the Karachay-Cherkess Republic, the average age of a generation is 26.98 years [7]. Therefore, the time for which the accumulation of the p.Arg261* variant occurred in the Karachay people is 275 ± 73 years. The mean year of birth of patients was 2005, so the period of the p.Arg261* variant spreading beginning falls at the beginning of the 18^th^ century (1730 ± 73 years).

## Discussion

### PKU and mild HPA incidence

The ratio of the identified patients with PKU and mild HPA is 54% and 46%, respectively, as determined by newborn screening data. Patients from Russia usually have mild HPA in less than 10% of cases. On one hand, such patients do not need dietary treatment and can lead a lifestyle that is close to that of healthy people. On the other hand, for an individual having two pathogenic variants in the *PAH* gene in a marriage between *PAH* pathogenic variant carriers, the probability of producing an affected offspring is 50% and the probability of a healthy carrier is 50%. In this case, the affected offspring may in turn have a severe clinical form of the disease. Based on these considerations, DNA diagnosis for patients with "mild HPA" should be observed by pediatricians and geneticists, and mutation carrier status should be determined in their future spouses with the subsequent possibility of prenatal DNA diagnostics. In families containing individuals with HPA, detailed explanations on the possible future pregnancies of females with HPA and the associated risks of maternal PKU should be provided.

### R261* haplotype origin

According to the literature, haplotype 3 in a German family and an incomplete haplotype in the Turkish family (MspI +, XmnI-) were described for the pathogenic variant p.Arg261* [20]. These alleles for the Turkish family coincide with those of the Karachays, but the data are insufficient to confirm the similarity of haplotypes. In Norway, the p.Arg261* variant has been detected on chromosomes with haplotype 1 [21], in Italy with haplotypes 1 and 4 [22, 23], and in Portugal with haplotype 4 [24]. Authors from Japan, who first described the variant p.Arg261*, identified the association of this variant with haplotype 2 [25]. In Iran, where the allele frequency of variant p.Arg261* is 4.9%, *PAH* mini-haplotypes have been analyzed (8/230) [26], and they did not match those of the Karachays. Nevertheless, it is obvious that the variant R261* has a common origin in the Karachays individuals studied.

### Time of p.Arg261* occurrence

**Fig 1:**
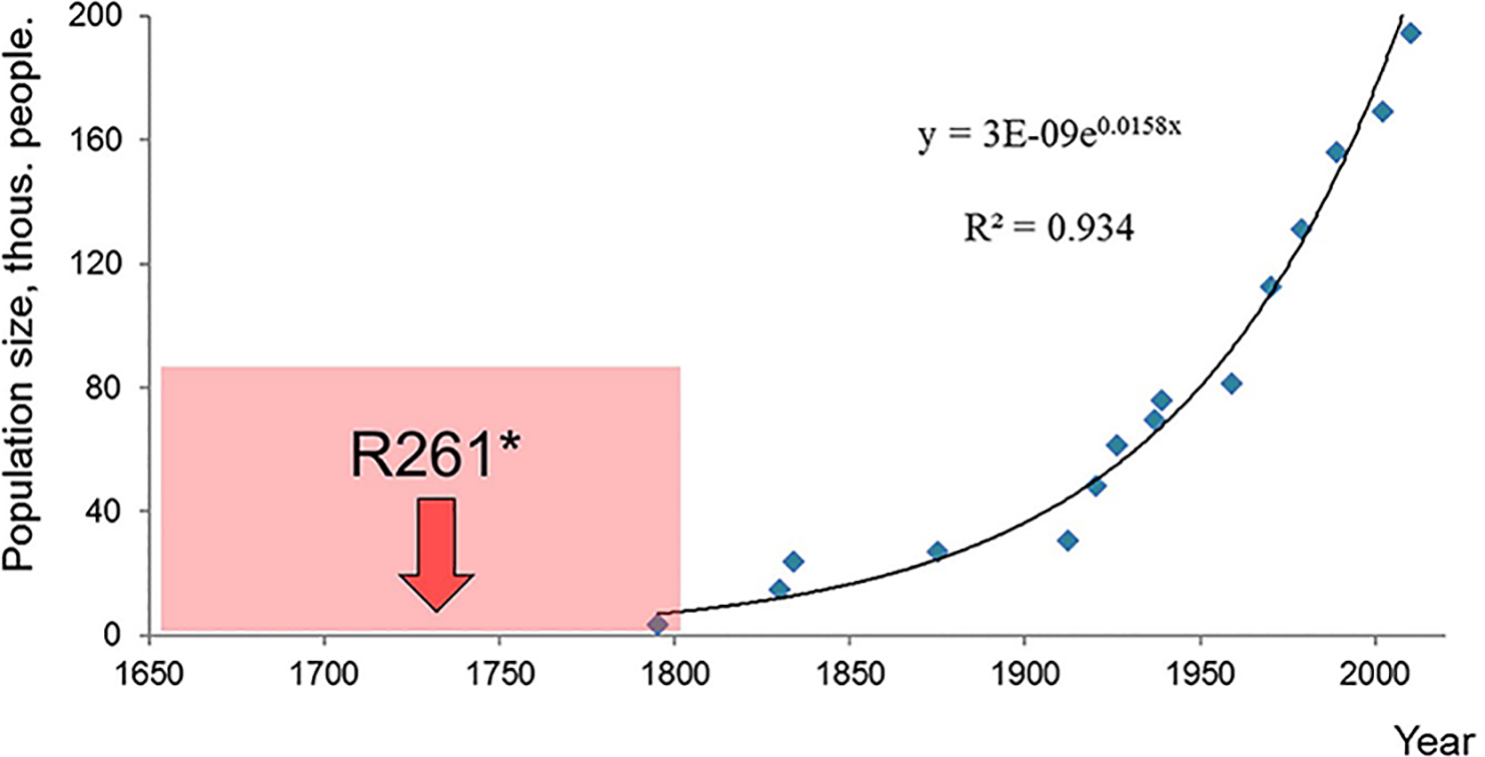
Karachay population dynamics from 1790 to 2010. The dots indicate the population numbers of the Karachay population [27]. The curve is the approximation of exponential dependence on the graph of abundance versus time. The point of p.Arg261* origin is marked by an arrow; the error interval is in a square.

Estimation of population growth parameters of Karachay people was carried out according to the dynamics of the Karachay population in the period from 1795 to 2010 [27]. The growth graph is shown in Fig 1. Using Microsoft Excel, exponential dependence was approximated with an approximate accuracy value of R^2^ = 0.934. The specific rate of population growth based on the graph is d = 0.0158. When calculating the correction g_0_ for a growing population, according to Labuda [28], g_0_ < 0 was obtained for all markers. Since the value of *d* characterizes the slope of the exponent and actually represents the growth rate of the isolated population size, in our case it can be said that the number of Karachays, although increasing exponentially, increased very slowly. The Karachay population cannot be characterized as rapidly growing, which is due to the apparently high resistance of the environment. Thus, we do not observe the effect of slowing down the genetic clock. The correction g_0_ turned out to be inapplicable and was not needed to calculate the mutation “age” in the Karachay population.

According to the literature, the first mention of Karachays occurred in the 12^th^ century. Until 1795, the population did not increase and was within 200 families. The growth phase began at the end of the 18th century, as shown in Fig 1. Given the estimated p.Arg261* mutation “age”, it can be assumed that the beginning of the variant distribution coincided with the onset of population growth in the Karachay population. As a result of the described processes, the investigated pathogenic variant spread widely among the Karachays population (1:16 healthy Karachays) in a relatively short time interval of 250–300 years.

## Conclusion

The DNA study of healthy Karachay-Cherkessia residents for the presence of frequent *PAH* pathogenic variants showed that the high incidence of HPA is caused by a significant frequency of *PAH* gene variants among Karachays. Among healthy Karachays, 1 in 16 is a carrier of the variant p.Arg261*, and 1 in 9 is a carrier of any variant of the *PAH* gene. The estimated incidence of all forms of the PAH-dependent HPA among Karachays is 1 in 332, and this the highest incidence reported to date. Thus, while Karachays constitute only 41% of the population of Karachay-Cherkessia, the wide distribution of PKU in this ethnic group accounts for the high incidence of PKU in the republic as a whole. The relatively high total frequency of mild *PAH* variants and the consequent high proportion of mild clinical forms of the disease (46% of patients identified by neonatal screening have mild HPA) probably played an important role in the process of accumulation of pathogenic variants among a healthy population.

The widespread distribution of p.Arg261* among Karachays suggested the presence of the founder effect. The study of *PAH* gene RFLP haplotypes on p.Arg261* chromosomes showed a common origin. The revealed haplotype, however, differs from all the described haplotypes for this variant, which suggested its independent origin. Investigation of the areas of tandem repeats in areas flanking the *PAH* gene made it possible to determine the disequilibrium in the linkage of these regions with the p.Arg261* variant and to calculate the time of its spread among the Karachays. The age of the mutation was g = 10.2 ± 2.7 generations or 275 ± 73 years. The variant p.Arg261* began to spread approximately 250–300 years ago in the population in the period corresponding to the beginning of exponential population growth.

## Supporting information

S1 Appendix *PAH* haplotype analysis. (DOCX)

S2 Appendix Allelic frequencies of flanking *PAH* gene STR markers on chromosomes with the mutation R261* (52 chromosomes) and among healthy Karachays (60 chromosomes). N–wild type alleles; R261*—R261* mutation carrying alleles; predominant alleles are marked in bold.
(DOCX)
