# Supplementary material for: Molecular-genetic causes for the high frequency of phenylketonuria in the population from the North Caucasus
Source: PLoS One. 2018 Aug 1;13(8):e0201489. doi: 10.1371/journal.pone.0201489 (PMC6070269; doi:10.1371/journal.pone.0201489)
Supplement: S1 Appendix — (DOCX) [file pone.0201489.s001.docx]

S1 Appendix. *PAH* haplotype analysis.

RFLP haplotype analysis via MLPA method.

S1.1 Table. The sequence of the oligonucleotides for the 1^st^ stage of the MLPA reaction.

| Oligonucleotide name | Oligonucleotide sequence |
| --- | --- |
| M1522296FG | CTCCATGCCAACAGTCGACATCGTTTATTTTCAGGAAGCACCAGCAGTCTTCG |
| M1522296FA | CTCCATGCCAACAGTCGACATCGTTTATTCTTTTCAGGAAGCACCAGCAGTCTTCA |
| M1522296R | GATCTCTTTCTCTGGAGGCCCAAATTCTTTATTCGATGCGATCCGATGCCTTCATG |
| M4646986FC | CTCCATGCCAACAGTCGACATCCTAACAGGGGAAGCAAATATTCTGAGC |
| M4646986FT | CTCCATGCCAACAGTCGACATCGATCTAACAGGGGAAGCAAATATTCTGAGT |
| M4646986R | AGCTGGGGAGGGAAGGCAGTTTATTCTTTTCGATGCGATCCGATGCCTTCATG |
| M4646987FC | CTCCATGCCAACAGTCGACATCCCTGCCTCAACCTCTTGAGC |
| M4646987FT | CTCCATGCCAACAGTCGACATCTCTCCTGCCTCAACCTCTTGAGT |
| M4646987R | AGCTGGGATTACAGGCATGCCTTCGATGCGATCCGATGCCTTCATG |
| M4646988FC | CTCCATGCCAACAGTCGACATCGTTTATTTCTCCTCCTTGTACCTCTGGTAGAATTC |
| M4646988FT | CTCCATGCCAACAGTCGACATCGTTTATTCTTGCTCCTCCTTGTACCTCTGGTAGAATTT |
| M4646988R | GGCTATGAATCCATCTGGTCCTGTTTATTCTTATTTCGATGCGATCCGATGCCTTCATG |
| M1042503FA | CTCCATGCCAACAGTCGACATCGTGTTTCCGCCTCCGACCTGTA |
| M1042503FG | CTCCATGCCAACAGTCGACATCTTTCCGCCTCCGACCTGTG |
| M1042503R | GCTGGCCTGCTTTCCTCTCGATGCGATCCGATGCCTTCATG |
| M1722383FC | CTCCATGCCAACAGTCGACATCCACAGGAATATTAGCTCTTCTGCCC |
| M1722383FT | CTCCATGCCAACAGTCGACATCTACCACAGGAATATTAGCTCTTCTGCCT |
| M1722383R | GGTACCCCACTGGGGATACTCAATTCGATGCGATCCGATGCCTTCATG |
| M869916FA | CTCCATGCCAACAGTCGACATCGTTTATTCTTATTGTTGGACTTTTGGAAGTGGAAGTGTTTA |
| M869916FC | CTCCATGCCAACAGTCGACATCGTTTATTCTTGTTGGACTTTTGGAAGTGGAAGTGTTTC |
| M869916R | GGGATTAGTTGAACTTTAACTGTAGAAGCATCTTCTTATTCGATGCGATCCGATGCCTTCATG |

S1.2 Table. The sequence of the oligonucleotides for the 2^nd^ stage of the MLPA reaction.

| Oligonucleotide name | Oligonucleotide sequence |
| --- | --- |
| Uni2 | CTCCATGCCAACAGTCGACATC |
| UniRMy | catgaaggcatcggatcgcatc |

S1.3 Table. The rs-numbers and names of the restriction sites; the length of the corresponding fragments.

| Restriction site | rs number (dbSNP) | Allele | Fragment length |
| --- | --- | --- | --- |
|  |  |  |  |
| XmnI | rs869916 | A | 126 |
|  |  | C | 123 |
| EcoRI | rs4646988 | T | 119 |
|  |  | C | 116 |
| BglII | rs1522296 | A | 112 |
|  |  | G | 109 |
| PvuIIa | rs4646986 | T | 105 |
|  |  | C | 102 |
| MspI | rs1722383 | T | 98 |
|  |  | C | 95 |
| PvuIIb | rs4646987 | T | 91 |
|  |  | C | 88 |
| AluI | rs1042503 | A | 85 |
|  |  | G | 82 |

Multiplex ligation-dependent probe amplification (MLPA) for detection of the *PAH* gene haplotypes was carried out in two stages. In the first stage, the original oligonucleotides (S1.1 Table) were annealed with the studied denatured DNA in the presence of a thermostable DNA ligase for 1 hour (t=64°C) in a volume of 5 μl of the reaction sweep of the following composition: 10-50 ng of genomic DNA; by 0.16-10 fmol / μl of each oligonucleotide (Eurogen, Russia); 0.4 units of Pfu-DNA ligase activity (Helicon, Russia), ligation buffer (20 mM Tris-HCl pH 7.5, 20 mM KCl, 10 mM MgCl2, 0.1% Igepal, 0.01 mM rATP, 1 mM DTT); 20 μl of mineral oil. At the second stage, a standard PCR (anneal t=66°C, 30 cycles) was performed with the universal oligonucleotides (S1.2 Table). A reaction mixture for PCR in a volume of 15 μl (0.25 μM each of each original oligonucleotide (Eurogen, Russia) was added to the mixture in which a ligase reaction was previously performed, 200 μM of each nucleoside triphosphate (Helicon, Russia); 1.0 unit of Biotaq DNA polymerase activity (Biomaster), PCR buffer (67 mM Tris-HCl, 16.6 mM (NH4) 2SO4, 0.01% Twin-20, pH 8.8.) The results of the reaction were visualized by electrophoresis in 9% of polyacrylamide gel (acrylamide : bis-acrylamide = 19 : 1).

STR and VNTR haplotypes were identified by PCR and Sanger sequencing with the oligonucleotides from the S1.4 Table.

S1.4 Table. The sequence of the oligonucleotides for the *PAH* STR and VNTR analysis.

| Oligonucleotide name | Oligonucleotide sequence |
| --- | --- |
| PAH STR F | CTGTGGAAAGCAGAAAGACC |
| PAH STR R | GTAATCATAAGTGTTCCCAGAC |
| PAH VNTR F | AGATTTTAATGTTCTCACCCGCC |
| PAH VNTR R | CTTGGAAACTTAAGAATCCCATC |
