# Supplementary material for: Molecular-genetic causes for the high frequency of phenylketonuria in the population from the North Caucasus
Source: PLoS One. 2018 Aug 1;13(8):e0201489. doi: 10.1371/journal.pone.0201489 (PMC6070269; doi:10.1371/journal.pone.0201489)
Supplement: S2 Appendix — N–wild type alleles; R261*—R261* mutation carrying alleles; predominant alleles are marked in bold. (DOCX) [file pone.0201489.s002.docx]

**S2 Appendix. Allelic frequencies of flanking *PAH* gene STR markers on chromosomes with the mutation R261* (52 chromosomes) and among healthy Karachays (60 chromosomes).**

| **STR** | **D12S1588** | | **D12S1727** | | **D12S78** | | **D12S338** | | **D12S317** | |
| --- | --- | --- | --- | --- | --- | --- | --- | --- | --- | --- |
|  | **R261*** | **N** | **R261*** | **N** | **R261*** | **N** | **R261*** | **N** | **R261*** | **N** |
| 1 |  | 3.3 |  |  | 3.8 | 10.0 | **94.2** | 18.3 |  | 31.7 |
| 2 | 9.6 | 6.7 |  |  |  | 10.0 | 5.8 | 28.3 |  | 11.7 |
| 3 | 17.3 | 40.0 |  |  | 1.9 | 1.7 |  | 25.0 | 7.7 | 13.3 |
| 4 | 9.6 | 23.3 |  |  |  | 5.0 |  | 5.0 | 7.7 | 8.3 |
| 5 | **63.5** | 13.3 |  |  |  | 8.3 |  | 11.7 |  | 5.0 |
| 6 |  | 11.7 |  |  |  | 10.0 |  | 3.3 |  | 3.3 |
| 7 |  | 3.3 | 7.7 | 13.3 |  | 6.7 |  | 1.7 |  | 6.7 |
| 8 |  | 1.7 | **76.9** | 21.7 | **92.3** | 1.7 |  | 3.3 |  | 5.0 |
| 9 |  |  |  | 15.0 |  | 18.3 |  | 1.7 |  |  |
| 10 |  |  | 1.9 | 11.7 |  | 3.3 |  |  |  | 6.7 |
| 11 |  |  | 13.5 | 3.3 |  | 8.3 |  |  |  |  |
| 12 |  |  |  | 3.3 | 1.9 | 6.7 |  |  |  |  |
| 13 |  |  |  | 6.7 |  | 3.3 |  |  | 1.9 | 1.7 |
| 14 |  |  |  | 6.7 |  |  |  |  | 5.8 | 3.3 |
| 15 |  |  |  | 3.3 |  | 6.7 |  |  | 3.8 |  |
| 16 |  |  |  |  |  |  |  |  | **73.1** |  |

N – wild type alleles; R261* - R261* mutation carrying alleles; predominant alleles are marked in bold.
